# Using prophylactic antioxidants to prevent noise-induced hearing damage in young adults: a protocol for a double-blind, randomized controlled trial

**DOI:** 10.1186/1745-6215-15-110

**Published:** 2014-04-05

**Authors:** Annick Gilles, Berina Ihtijarevic, Kristien Wouters, Paul Van de Heyning

**Affiliations:** 1 Department of Translational Neuroscience, Faculty of Medicine, University of Antwerp, Antwerp, Belgium; 2 Department of Head and Neck Surgery, Antwerp University Hospital, Wilrijkstraat 10, 2650 Edegem, Belgium; 3 Department of Medical Management, Statistical Analysis, Antwerp University Hospital, Edegem, Belgium

**Keywords:** Noise-induced hearing loss, Antioxidants, *N*-acetylcysteine, Magnesium, Adolescents, ROS, RNS, Prevention, Noise damage, Randomized controlled trial

## Abstract

**Background:**

During leisure activities young people are often exposed to excessive noise levels resulting in an increase of noise-induced symptoms such as hearing loss, tinnitus and hyperacusis. Noise-induced tinnitus is often perceived after loud music exposure and provides an important marker for overexposure as a temporary threshold shift that is often not experienced by the individual itself. As oxidative stress plays an important role in the pathogenesis of noise-induced hearing loss, the use of antioxidants to prevent hearing damage has recently become the subject of research.

**Methods:**

This study proposes a randomized, double-blind, placebo-controlled crossover trial to assess the effects of a prophylactic combination of *N*-acetylcysteine (600 mg) and magnesium (200 mg) prior to leisure noise exposure in young adults. The primary outcome measure is the tinnitus loudness scored by a visual analogue scale (VAS). Secondary outcome measures are the differences in audiological measurements for the antioxidant treatments compared to placebo intake. Audiological testing comprising of pure tone audiometry including frequencies up to 16 kHz, distortion product otoacoustic emissions, transient-evoked otoacoustic emissions and speech-in-noise testing will be performed prior to and within 7 hours after noise exposure. By use of a mixed effects statistical model, the effects of antioxidants compared to placebo intake will be assessed.

**Discussion:**

As adolescents and young adults often do not use hearing protection while being exposed to loud music, the use of preventive antioxidant intake may provide a useful and harmless way to prevent noise-induced hearing damage in this population. Furthermore, when exposed to hazardous noise levels the protection provided by hearing protectors might not be sufficient to prevent hearing damage and antioxidants may provide additive otoprotective effects. Previous research mainly focused on occupational noise exposure. The present study provides a protocol to assess the usefulness of antioxidants during leisure noise activities.

**Trial registration:**

The present protocol is registered at ClinicalTrials.gov: NCT01727492.

## Background

Adolescents and young adults frequently expose themselves to hazardous noise levels during social events or through personal listening devices. This has led to an increase of noise-induced symptoms such as noise-induced hearing loss (NIHL) and noise-induced tinnitus (NIT) over the last few years [1,2]. Although 15% to 18% of the young population already experiences permanent NIT and the majority acknowledge the fact that loud music can damage the hearing, the use of hearing protection remains very low [3-5].

Oxidative stress plays a crucial role in the pathogenesis of NIHL and NIT. During excessive noise exposure, the outer hair cells endure metabolic depletion leading to accumulation of reactive oxygen species (ROS) and reactive nitrogen species (RNS) which may ultimately lead to necrosis and apoptosis [6]. While necrosis is a passive form of cell death, usually occurring after gross physical or chemical insult, associated with cell swelling and eventually causing cell rupture and loss of function, apoptosis is an active way of cell death which also occurs under normal metabolic circumstances [7]. However, when apoptosis is ‘forced’ (for example as a consequence of noise exposure) and by this initiated at the wrong time, crucial healthy outer hair cells may die [7].

Under normal circumstances, the human cochlea contains molecules including vitamins, glutathione, enzymes and reactive transcription, which work together to form a complex and sophisticated defense mechanism against oxidative molecules [8]. In cases of excessive noise exposure, the naturally occurring antioxidant systems may not render sufficient detoxifying effects, leading to the possible important role of antioxidant treatment after acoustic trauma [9,10].

While antioxidant treatment for noise-induced hearing damage has often been the subject of animal research [11-17], well-performed human randomized controlled trials remain scarce. Examining the literature, two antioxidants have been mainly investigated as prophylactic prevention in animal and also some human studies: *N*-acetylcysteine (NAC) [18-21] and magnesium (Mg^2+^) [22,23]. As oxidative stress triggers several cascades, it has been proven that a combination of antioxidants may provide better effects compared to a single antioxidant [24]. Most human antioxidant studies have mainly investigated the effects during occupational noise exposure [25]. Hence, the use of prophylactic antioxidants during recreational noise is unclear. As the use of hearing protection does not always render sufficient protection while exposed to hazardous noise levels, an addition of antioxidants may reduce noise-induced symptoms in young people.

The present article proposes a protocol for a clinical study consisting of a double-blind, randomized, placebo-controlled trial with a combination of NAC and Mg^2+^ as prophylactic antioxidants in adolescents during leisure noise exposure. The present report will follow the guidelines expressed by Consolidated Standards of Reporting Trials (CONSORT).

## Design

This study proposes a randomized, double-blind, placebo-controlled crossover trial. Participants need to spend at least 3 consecutive hours in an environment where loud music is played (>95 dB(A)) after taking either a combination of antioxidants (NAC and Mg^2+^) or a placebo, 1 hour prior to noise exposure. Each participant needs to repeat this procedure four times (interval between trials at least 4 days) and each participant will receive twice the antioxidants as well as the placebo. This study aims to investigate the combined effects of NAC and Mg^2+^ by use of four repeated treatments on the hearing of noise-exposed young adults.

### Recruitment

We are recruiting students by advertisements sent by email and distributing information sheets around the University of Antwerp (Antwerp, Belgium). The aim is to always investigate groups (at least two individuals) of peers going out together and therefore attending the clinic within the time range of 7 hours after noise exposure. It is assumed that the pressure of peers also participating in the study will reduce the risk of drop-out. In addition, at the stage of enrolment it will be determined which occasions the subjects will attend and test moments will be scheduled. The aim is to plan all treatments within the range of 3 months in order to reduce the risk of drop-out.

### Treatment protocol

Participants will receive an antioxidant combination of NAC and Mg^2+^, or a placebo drug (sugar pill). The antioxidant packets contain two pills of each 300 mg NAC and 100 mg Mg^2+^. Both pills, providing a total dose of 600 mg NAC and 200 mg Mg^2+^, should be taken orally 1 hour before noise exposure with a large glass of water. In 50%, the packets contain a placebo with an identical appearance and weight as the antioxidants. All participants (as well as the researchers) will be blinded to the order of antioxidants and placebo. The effects of NAC and Mg^2+^ at the respective doses of 600 mg and 200 mg will be analyzed as well as the effects of the combined product, for which no significant adverse effects have been reported in the past. Adverse events are defined as symptoms occurring after the administration of antioxidants or placebo that were not necessarily related to the intervention. Participants will receive a telephone number to report adverse events during the study and will be urged to come to the clinic in case of serious adverse events.

### Permitted and prohibited concomitant treatments

The use of alcohol and tobacco is limited to a maximum of two alcohol consumptions and no to two cigarettes. Other psychoactive drugs are strictly prohibited.

### Inclusion criteria

The inclusion criteria are as follows:

• Age between 18 and 28 years old (males as well as females).

• Experience of temporary tinnitus after leisure noise exposure in the past scoring at least 5 or more on a visual analogue scale (VAS) for loudness.

• Willing to attend an indoor/outdoor music event with mean noise levels ≥95 dB(A) LAeq, 60 min for at least 3 consecutive hours.

### Exclusion criteria

The exclusion criteria are as follows:

• Middle ear pathologies such as otitis media and the perforation of the tympanic membrane and history of such pathologies.

• Known allergies for NAC or Mg^2+^.

• Adolescents using hearing protection.

• Recreational noise exposure within 4 days prior to a study trial.

### Randomization

The production of the antioxidant and placebo packets as well as the randomization of the ABAB protocol will be performed by an independent pharmacist. The packets of antioxidants and placebos will be labeled with a number corresponding with the numbers on the forms. The randomization file will be put into a sealed envelope and retained in a safe at the pharmacy. The randomization table will not be available for assessment by anyone else involved in the study. At the end of the study the envelope will be requested.

### Sample size calculation and statistical analyses

A power calculation was performed in order to make an estimation of the sample size needed to detect significant differences in the VAS between the experimental and control group by use of the software G-Power. A two-sided paired *t*-test for mean differences was performed in which an α-level of 0.05 and a nominal power of 80% were used. Assuming a minimal difference of clinical effect of 0.5 between the pre- and post-noise measurements on the VAS, a non-parametric calculation showed a requirement of 21 participants.

All data in this trial will be assessed with SPSS Statistics version 20.0 (SPSS, Chicago, IL, USA) and SAS version 9.1.3 (SAS, Cary, NC, USA). Because multiple correlated measurements will be performed in the same participants, a mixed effects model will be applied. This analysis is preferable over more traditional approaches such as repeated measures analysis of variance (ANOVA) because of the advantages to deal with missing values. The main analysis will be fully specified before unmasking.

### Outcomes

The primary outcome measure will be the score on the VAS for tinnitus loudness, which will be measured pre-noise exposure (at the time of antioxidant intake) and 1 hour after noise exposure. The assumed expectation is a drop of 50% of the tinnitus loudness in the antioxidant group compared to the placebo group.

The secondary outcome measure is the evaluation of a significant difference in test results of the audiological testing (audiometry, otoacoustic emissions and speech-in-noise tests) when comparing the placebo results to the antioxidant trials.

A series of audiological tests (described in the following paragraphs) will be performed prior to and within 7 hours after each noise exposure.

#### Main outcome measure

##### Visual analogue scale (VAS)

Tinnitus loudness will be scored on a VAS from 0 to 10 (0, no tinnitus at all; 10, extremely loud tinnitus, could not possibly be louder), once at the time of antioxidant (or placebo) intake (1 hour prior to exposure) and once 1 hour after noise exposure. At both occasions the VAS will be undertaken in a quiet room.

#### Secondary outcome measures

##### Pure tone audiometry

Pure tone liminar audiometry will be performed according to the current clinical standards (International Organization for Standardization (ISO) 8253-1:2010) using a two-channel AC40 audiometer (Interacoustics, Assens, Denmark) in a silent room. Air conduction thresholds will be measured under headphones at 125 Hz, 250 Hz, 500 Hz, 1 kHz, 2 kHz, 3 kHz, 4 kHz, 6 kHz and 8 kHz. In addition, high frequency air conduction thresholds will be performed including 9 kHz, 10 kHz, 11,200 Hz, 12,500 Hz, 14 kHz and 16 kHz. When air conduction thresholds between 250 Hz and 4 kHz exceed normality levels of 20 dB HL, the bone conduction threshold will be measured on 250 Hz, 500 Hz, 1 kHz, 2 kHz, 3 kHz and 4 kHz in order to make a distinction between conductive and sensorineural hearing loss.

##### Otoacoustic emissions

Transient-evoked otoacoustic emissions (TEOAEs) as well as distortion-product otoacoustic emissions (DPOAEs) will be performed. TEOAEs are elicited with click sounds presented at an intensity level of 80 dB SPL and recorded over a frequency range of 500 to 4,000 Hz. DPOAEs are elicited by use of a set of two pure tone frequencies (f1 and f2) closely spaced and presented simultaneously at a level of 55 dB SPL for f1 and 65 dB SPL for f2. The largest and most robust distortion product is 2f1-f2 and can be detected in almost all normal ears. The amplitude of 2f1-f2 is optimized by use of the frequency ratio f2/f1 = 1.22. DPOAEs will be recorded in the frequency region from 1 kHz to 6 kHz.

##### Speech-in-noise testing

Speech-in-noise testing will be performed using the Leuven intelligibility sentence test (LIST) [26] in an adaptive procedure with noise at a fixed level of 65 dB SPL. Each list consists of ten sentences. Both ears will be tested separately by use of headphones. The procedure starts at a signal-to-noise ratio (SNR) of 0 dB meaning that speech and noise are presented with an equal loudness (65 dB SPL). Subsequently, the intensity level within a list of sentences varies in steps of 2 dB adaptively in a 1-down (when the keywords in the sentence are correctly repeated), 1-up procedure to determine the 50% correct identification point, which is called the speech reception threshold (SRT), expressed in dB SNR. Three conditions will be tested for both ears: sentences in steady-state noise, sentences in amplitude-modulated noise by a 10 Hz modulation and sentences in amplitude-modulated noise by a 15 Hz modulation.

##### Controlling the noise levels

In order to be able to respect the inclusion criterion of the minimal noise levels of 95 dB(A) LAeq, 60 min for 3 consecutive hours, all participants will be equipped with portable decibel meters (CEL350, Casella, Bedford, UK). The decibel meter can be fixed onto a shirt or trousers but participants must be aware that the microphone entrance should remain free from overhanging clothing.

### Ethics

Written consent will be obtained from each participant. This study protocol was approved by the ethical committee at Antwerp University Hospital in June 2012 with protocol number 12/18/172.

### Dissemination policy

According to the Standard Protocol Items: Recommendations for Interventional Trials (SPIRIT) guidelines, the authors declare that data that break the blind will not be presented prior to the release of mainline results. The breaking of the blind will occur at the end of the study. A clinical article will be written on the primary (including also secondary) outcomes of the study and results will be disseminated regardless of the magnitude or direction of effect. The present trial is not industry-initiated. As such, there are no publication restrictions imposed by sponsors.

In addition, a full study report, anonymized participant-level dataset and statistical code for generating the results will be made publicly available no later than 3 years after the termination of the study for sharing purposes.

## Discussion

As adolescents and young adults often do not use hearing protection while being exposed to loud music, the use of preventive antioxidant intake may provide a useful and harmless way to prevent noise-induced hearing damage in this population. Furthermore, when exposed to hazardous noise levels the protection provided by hearing protectors might not be sufficient to prevent hearing damage and antioxidants may provide additive otoprotective effects. Previous research mainly focused on occupational noise exposure. The present study will assess the usefulness of antioxidants during leisure noise activities.

The authors acknowledge the limitation of not including blood samples in the present protocol in order to control for naturally varying Mg^2+^ levels in the individual. However, the investigators considered that it was very likely that the drop-out rate would be very high when participants needed to give a blood sample at every test moment, which would be eight in total. It is also assumed that it is the acute increase of NAC and Mg^2+^ prior to noise exposure that provides the extra otoprotective effects and not so much the naturally high or low levels.

To our knowledge, this study is the first to combine NAC and Mg^2+^ as prophylactic antioxidants for noise-induced tinnitus and noise-induced hearing damage in young people performed in a randomized, placebo-controlled trial. Furthermore, both participants and investigators will be blinded for the sequence of antioxidants and placebo. Thus, this would not influence on one hand the participants in rating the tinnitus loudness and on the other hand the investigators in performing the audiological testing, limiting investigator bias as far as possible.

## Trial status

To date, half of the needed participants have been included in the study. The procedure started in September 2012. The study is anticipated to be completed in April 2014.

## Abbreviations

ANOVA: Analysis of variance; CONSORT: Consolidated Standards of Reporting Trials; DPOAE: Distortion-product otoacoustic emission; LIST: Leuven intelligibility sentence test; Mg2+: Magnesium; NAC: *N*-acetylcysteine; NIHL: Noise-induced hearing loss; NIT: Noise-induced tinnitus; RNS: Reactive nitrogen species; ROS: Reactive oxygen species; SNR: Signal-to-noise ratio; SPIRIT: Standard Protocol Items: Recommendations for Interventional Trials; SRT: Speech reception threshold; TEOAE: Transient-evoked otoacoustic emission; VAS: Visual analogue scale.

## Competing interests

The authors declare that they have no competing interests in the publication of the present protocol.

## Authors’ contributions

AG conceived and designed the study, and undertook data collection and analysis, manuscript writing and final approval of the manuscript. BI conceived and designed the study, and undertook data collection and analysis, manuscript writing and final approval of the manuscript. KW undertook data collection and analysis, critical revision and final approval of the manuscript. PvdH conceived and designed the study, and undertook manuscript writing and final approval of the manuscript.

## Authors’ information

AG is a third year PhD student at the University of Antwerp. BI is a seventh year medical student at the University of Antwerp. KW is a PhD statistician at the University Hospital Antwerp. PvdH is Head of the Department of Otorhinolaryngology and Head and Neck Surgery at the University Hospital Antwerp and Professor at the University of Antwerp.
